# Surimi Gel Quality Enhanced by κ-Carrageenan/Konjac Glucomannan and Sodium Caseinate-Stabilized Emulsion

**DOI:** 10.3390/gels12100907

**Published:** 2026-10-08

**Authors:** Tianzhen Xiong, Lala Li, Guanchen Wu, Lihua Zhang, Wei Xu

**Affiliations:** 1 College of Tea and Food Science, Xinyang Normal University, Xinyang 464000, China; tzxiong@xynu.edu.cn; 2 College of Life Science, Dabie Mountain Laboratory, Xinyang Normal University, Xinyang 464000, China; 15517984382@163.com (L.L.); w924854122@163.com (G.W.)

**Keywords:** surimi gel, konjac glucomannan, κ-carrageenan, sodium caseinate-stabilized camellia oil emulsion, gel properties

## Abstract

This study investigated the effects of a combination of κ-carrageenan (CRG), konjac glucomannan (KGM) and a sodium caseinate-stabilized camellia oil emulsion on the quality attributes of surimi. The results demonstrated that adding the emulsion alone enhanced the textural properties but failed to increase the water-holding capacity (WHC) or reduce cooking loss. In contrast, incorporating both polysaccharides with the emulsion reduced cooking loss to 7.3% of that of the control (2.3 ± 1.0% vs. 31.4 ± 1.4%) and increased the WHC to 97.7 ± 1.3%, representing a 47.8% improvement over the control (66.1 ± 1.2%). Furthermore, this ternary composite system yielded substantial enhancements in the textural profile of the gels. These improvements were attributed primarily to the formation of hydrogen bonds between the polysaccharides and surimi proteins, which strengthened intermolecular interactions. Microstructural observations corroborated this mechanism, revealing that the composite system promoted the formation of a denser and more uniform network, which improved the freeze–thaw stability of the surimi. Sensory evaluation indicated that the combined treatment enhanced the flavor profile and acceptability of surimi gels. Thus, KGM and CRG can be combined with sodium caseinate-stabilized camellia oil emulsion to improve the quality of surimi gels. These results offer valuable theoretical guidance for advancing surimi-based foods.

## 1. Introduction

Surimi is a concentrated fish protein product manufactured through processes such as meat collection, rinsing, dewatering, and refining. This food ingredient is highly valued worldwide for its nutritional benefits, delicate flavor, and cost-effectiveness [1]. The quality of surimi and its derived products is primarily assessed by flavor attributes and gelation properties. However, the rinsing step in surimi production often results in considerable lipid loss, thus negatively impacting both flavor and nutritional value [2]. Additionally, conventional freshwater surimi products face several challenges—including high cooking loss, suboptimal gel properties, poor freeze–thaw stability, and quality deterioration during cold chain logistics—which limit their broader application in the food industry [3,4]. Consequently, the use of natural additives to synergistically optimize the nutritional and structural characteristics of surimi gels, while enhancing sensory properties and maintaining processing performance, is critical for advancing the freshwater surimi industry [5].

To address these challenges, researchers have explored strategies involving raw material pretreatment, innovative processing techniques, and the addition of exogenous ingredients to improve surimi quality. In recent years, natural polysaccharides and emulsions have attracted growing interest for their potential to enhance surimi gels. Appropriate incorporation of polysaccharides can improve gelation capacity, increase water-holding capacity (WHC), modify texture, delay lipid and protein oxidation, and enhance product stability. For example, the addition of insoluble dietary fibers increases gel strength, elasticity, and deformation resistance, while 1% soluble dietary fiber can significantly enhance the overall performance of surimi gels [6]. Sun et al. demonstrated that onion stalk polysaccharides mitigate the negative impact of fish oil on surimi gelation [7]. This effect subsequently enhances the quality and storage stability of frozen surimi. Similarly, the incorporation of flaxseed gum has been shown to improve the physicochemical properties of surimi and serve as a fat substitute, facilitating the development of low-fat surimi products [8]. However, using a single polysaccharide often has limited efficacy for modulating surimi properties. For instance, although κ-carrageenan (CRG), hydroxypropylated cassava starch, and curdlan each enhance the texture and WHC of surimi gels, their combined application yields a synergistic effect, resulting in more pronounced quality improvements [9]. CRG, an anionic polysaccharide derived from red seaweed, exhibits excellent gelling, thickening, and water-binding properties. Studies indicate that CRG accelerates the aggregation of myofibrillar proteins (MP) and promotes the formation of a dense, fine three-dimensional (3D) network through ionic bonds, disulfide bonds, and hydrophobic interactions, thereby improving WHC and gel strength [10]. The incorporation of CRG can enhance the quality of low-salt surimi and increase saltiness perception [11,12]. Konjac glucomannan (KGM), a neutral polysaccharide soluble in water, is derived from konjac tubers. It exhibits significant thickening, swelling, and heat-induced gelling properties, along with various physiological benefits such as cholesterol-lowering, anti-obesity, and anti-tumor activities [13]. Studies have shown that KGM stabilizes MP conformation and compensates for structural defects in single-protein gels [14]. The addition of KGM or its derivatives significantly enhances the gel properties, freeze–thaw stability, and edibility of surimi [15,16]. Moreover, both KGM and CRG can also serve as fat replacers, imparting an oily texture to meat products, and are widely employed in product reformulation. Previous work has established that KGM and CRG can form interpenetrating network gels via intermolecular entanglement and hydrogen bonding [17,18]. However, the combined use of KGM and CRG to enhance the gel properties and quality of freshwater surimi has not yet been fully explored.

To improve the flavor, taste, and nutritional value of surimi products, animal fats or vegetable oils are often added [19]. It is noteworthy that animal fats are rich in cholesterol and saturated fatty acids. A diet with excessive amounts of these components is linked to a heightened risk of obesity and cardiovascular disease. In contrast, liquid vegetable oils are rich in unsaturated fatty acids and are generally regarded as healthier lipid supplements for surimi gels [20,21]. However, the direct addition of vegetable oils may disrupt the gel network of surimi, resulting in inferior texture [22]. Previous findings have demonstrated that increasing the content of vegetable oils (e.g., rapeseed, corn oil) in surimi reduces gel breaking force, storage modulus, and loss modulus, despite improvements in flavor and color [23]. This has been attributed to an imbalance between oil-water interfacial tension and protein adsorption affinity [24]. Pre-emulsifying liquid oils with food-grade stabilizers has been found to promote oil incorporation and stability, minimize structural disruptions from oil-protein interactions, and foster the formation of denser protein networks. For example, the use of collagen peptide-stabilized fish oil emulsions restores lipid content in surimi, improves freeze–thaw stability, and reduces cooking loss [25]. Mi et al. demonstrated that starch-based peanut oil emulsions positively affect the gel strength, WHC, texture, whiteness, and sensory attributes of surimi gels [26]. However, the quality of emulsion-filled surimi gels is significantly affected by formulation factors such as the choice of emulsifier, the type and concentration of oil used, and the fatty acid composition of the oil. Sodium caseinate (CAS), an amphiphilic milk-derived emulsifier, displays outstanding emulsifying and gelation properties. Xu et al. showed that CAS-stabilized diacylglycerol emulsions facilitate the development of dense gel networks in surimi, significantly enhancing gel strength and improving odor attributes [27]. Moreover, emulsions co-stabilized by CAS/tannic acid complexes have been shown to improve the 3D printing accuracy of surimi gels [28]. Camellia oil, a health-promoting edible oil recommended by the FAO, is rich in unsaturated fatty acids, polyphenols, vitamin E, phytosterols, and sasanquasaponin. It also exhibits pharmacological effects such as anti-inflammatory, hypolipidemic, and hepatoprotective activities [29]. However, the application of CAS-stabilized camellia oil emulsions in surimi products has received limited investigation. Polysaccharides can facilitate the adsorption of CAS at the oil–water interface, increase interfacial film thickness, and reduce droplet size, thereby improving emulsion stability [30,31]. Although the effects of KGM, CRG, and CAS-stabilized emulsions on surimi quality have attracted considerable attention [3,19,28], the ternary combination of KGM and CRG with a CAS-stabilized camellia oil emulsion has received little attention in recent studies.

This study evaluated the effects of individual KGM and CRG, CAS-stabilized camellia oil emulsions, and their combinations on the WHC, cooking loss, textural properties, freeze–thaw stability, and sensory acceptability of surimi gels. Additionally, microstructural and rheological analyses were conducted to elucidate the mechanisms underlying the effects of polysaccharides and emulsions on surimi gelation. These findings establish a theoretical foundation for the formulation of surimi products that exhibit an optimal balance among nutritional value, sensory characteristics, and processing performance.

## 2. Results and Discussion

### 2.1. WHC and Cooking Loss of Surimi Gels

**Figure 1:**
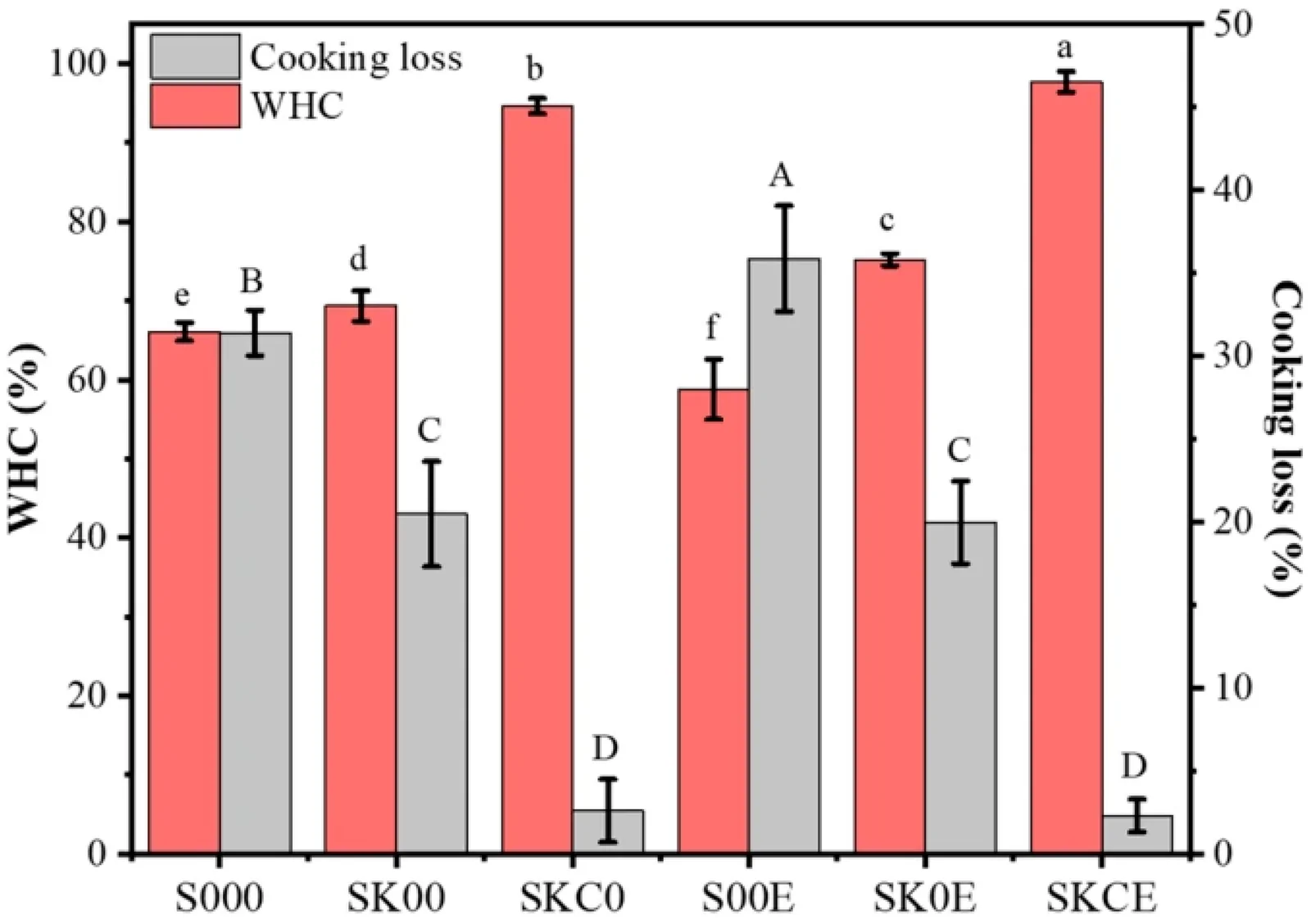
Water holding capacity and cooking loss of surimi gels added with polysaccharides, CAS-stabilized camellia oil emulsion, and their combinations. Different capital and lowercase letters indicate significant differences (*p* < 0.05).

WHC characterizes a sample’s ability to retain moisture and is a critical indicator of food acceptability [32]. Figure 1 shows the effects of polysaccharides, CAS-stabilized camellia oil emulsion, and their combinations on the WHC of surimi gels. S000 (control sample) exhibited a relatively low WHC (66.1%), attributed to the weak self-supporting ability of the surimi gel, whose structure is susceptible to deformation or damage under centrifugal force [33]. Incorporation of KGM into surimi increased the WHC by 4.8%, reaching 69.3%. When both KGM and CRG were added, the WHC further increased to 94.6%. This improvement is likely attributable the ability of polysaccharide molecules to form cross-link with proteins, which reduces water channels and microcracks within the gel matrix, thereby limiting moisture loss during centrifugation [34]. Furthermore, these polysaccharides can not only fill network pores but also cross-link via intermolecular forces and hydrogen bonding, enhancing the compactness and stability of the surimi gel network and reducing water loss.

Protein–protein interactions are major determinants of gel network formation and WHC. The incorporation of emulsion to surimi resulted in an 11% reduction in WHC compared with the control (S00E vs. S000). This may be attributable to interactions between MP and CAS within the emulsion, which weaken both the emulsion droplet interface and the protein–protein interactions among MP, resulting in a loose gel network and reducing WHC. However, the addition of polysaccharides after the emulsion significantly increased WHC (SK0E, SKCE vs. S00E). When KGM, CRG, and emulsion were incorporated simultaneously, sample SKCE reached a WHC of 97.7%, significantly higher than other samples. Here, the polysaccharides may synergistically interact with CAS to stabilize the emulsion interface, and cross-link both with one another and with surimi proteins, generating a more ordered, compact network that effectively entraps moisture and improves WHC.

Cooking loss, which reflects the matrix’s ability to retain moisture and oil, is similarly influenced by gel microstructure. Generally, lower cooking loss indicates a denser and more ordered gel network [27]. Addition of emulsion alone increased the cooking loss by 14.3% over the control (S000), reaching 35.9% (Figure 1). Large emulsion droplets can increase the distance between MP molecules, impede cross-linking, and thus degrade gel structure, reducing capacity for water and oil retention [35]. When the high-phase emulsion was incorporated into the surimi gel, a similar result was observed [36]. Addition of KGM significantly decreased cooking loss, and the lowest cooking loss (2.3%) was observed in the sample with simultaneous incorporation of KGM, CRG, and emulsion. In the meat industry, cooking losses of 1–5% are generally acceptable. Hence, surimi gels combined with KGM, CRG, and camellia oil emulsion are more suitable for food processing applications.

### 2.2. Textural Analysis of Surimi Gels

**Figure 2:**
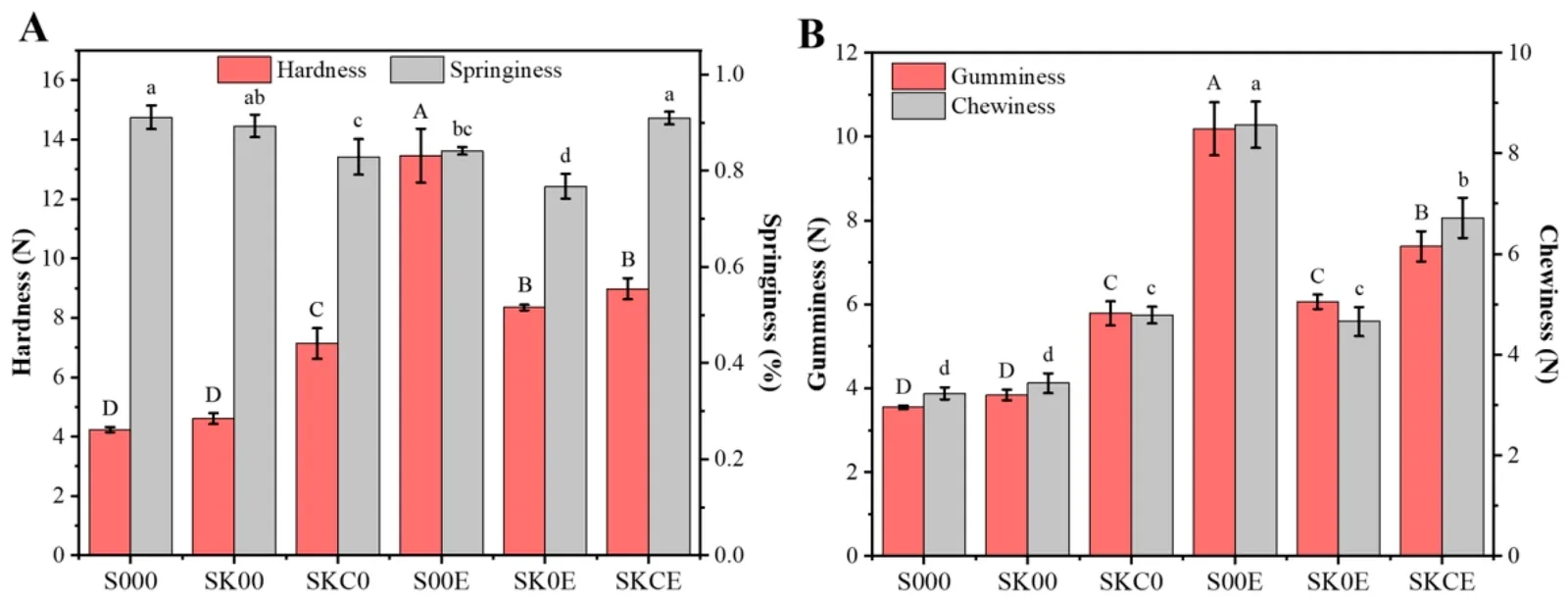
Hardness and springiness (**A**), gumminess and chewiness (**B**) of surimi gels. Different capital and lowercase letters indicate significant differences (*p* < 0.05).

Texture properties such as hardness, springiness, gumminess, and chewiness reflect the strain behavior of surimi gel under compression, serving as important indicators of gel structure and sensory quality. As shown in Figure 2, the addition of KGM (or KGM and CRG together) increased the hardness, gumminess, and chewiness of the surimi gels while reducing their springiness. Polysaccharides can be incorporated into the MP gel network and interact with proteins via hydrogen bonds and electrostatic interactions, thereby stabilizing the gel structure and enhancing its hardness [7]. The highest hardness, gumminess, and chewiness were observed in the sample containing camellia oil emulsion alone (S00E). This result may be attributed to the disruption of the emulsion structure during gelation, particularly under the high-temperature preparation conditions, which promotes water and oil loss. The consequent reduction in water content increases the relative solid content and produces a more compact internal matrix, thereby enhancing gel hardness. Because chewiness is positively correlated with hardness [37], it increased accordingly. Compared with samples lacking emulsion (S000, SK00, SKC0), those containing camellia oil emulsion (S00E, SK0E, SKCE) displayed enhanced texture properties, highlighting the beneficial synergistic effects of polysaccharides and emulsions on surimi gel properties. Previous studies have shown that the incorporation of polysaccharide–emulsion complexes improves matrix uniformity and densifies the gel structure, thereby improving surimi quality [38]. These findings are consistent with the results of the present study.

### 2.3. FTIR Analysis of Surimi Gels

**Figure 3:**
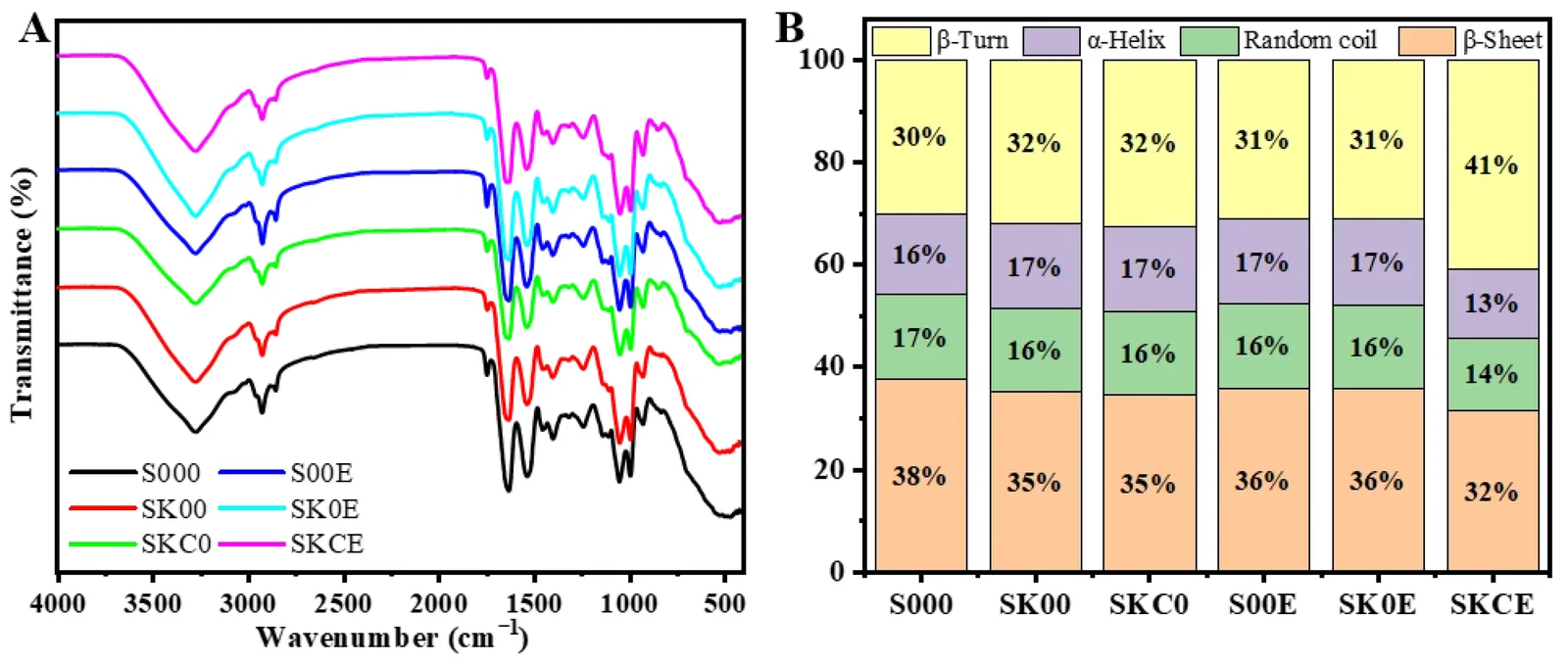
FTIR spectra (**A**) and secondary structure fractions (**B**) of surimi gels.

FTIR spectroscopy provides insight into intermolecular and intramolecular interactions in gels, including changes in protein secondary structure. As shown in Figure 3A, the FTIR spectra of surimi gels, regardless of whether polysaccharides, emulsions, or both were added, featured similar characteristic absorption peaks, with no new peaks observed. This finding suggests that no new types of covalent chemical bonds were formed within the gel system. Disulfide bonds, hydrogen bonds, and electrostatic interactions are intrinsic forces involved in surimi gelation [10]. Therefore, the absence of new absorption peaks indicates that polysaccharides and emulsions interact with the surimi primarily through noncovalent interactions. The increased absorption intensity in the 1180–953 cm^−1^ following polysaccharide addition reflects enhanced adhesion between MP and polysaccharide molecules. Changes in protein secondary structure showed that the α-helix, β-sheet, and β-turn contents in the control (S000) were 16%, 38%, and 30%, respectively (Figure 3B). Reactive groups originating from interfacially adsorbed proteins in the emulsion and from the polysaccharides may participate in intermolecular cross-linking during thermal gelation, thereby regulating the higher-order structure of MP molecules [14]. Nevertheless, the addition of either the emulsion or the polysaccharides alone did not significantly alter the relative proportions of the secondary-structure components in the resulting surimi gels (SK00 and SKC0 vs. S000; S00E vs. S000). Previous studies have shown that incorporating polysaccharides or Pickering emulsions into protein-based gel typically decreases the relative proportion of α-helix structures while increasing the β-sheet content [39,40]. The discrepancy between these reports and the present findings may be attributed to differences in the physicochemical properties of the polysaccharides and interfacial proteins, which lead to distinct interaction behaviors during MP gel formation [15]. However, the combined addition of the CAS-stabilized camellia oil emulsion and both polysaccharides resulted in an increase in β-turn (41%), decreased β-sheet (32%), and slight reductions in α-helix (13%) and random coil (14%) contents. These changes may be due to the influence of additives on the microenvironment of the gel matrix. Oil droplets interact with hydrophobic amino acid residues on MP, leading to the exposure of additional hydrophobic groups and inducing conformational changes in the protein structure.

### 2.4. Temperature Sweep Analysis of Surimi Gels

**Figure 4:**
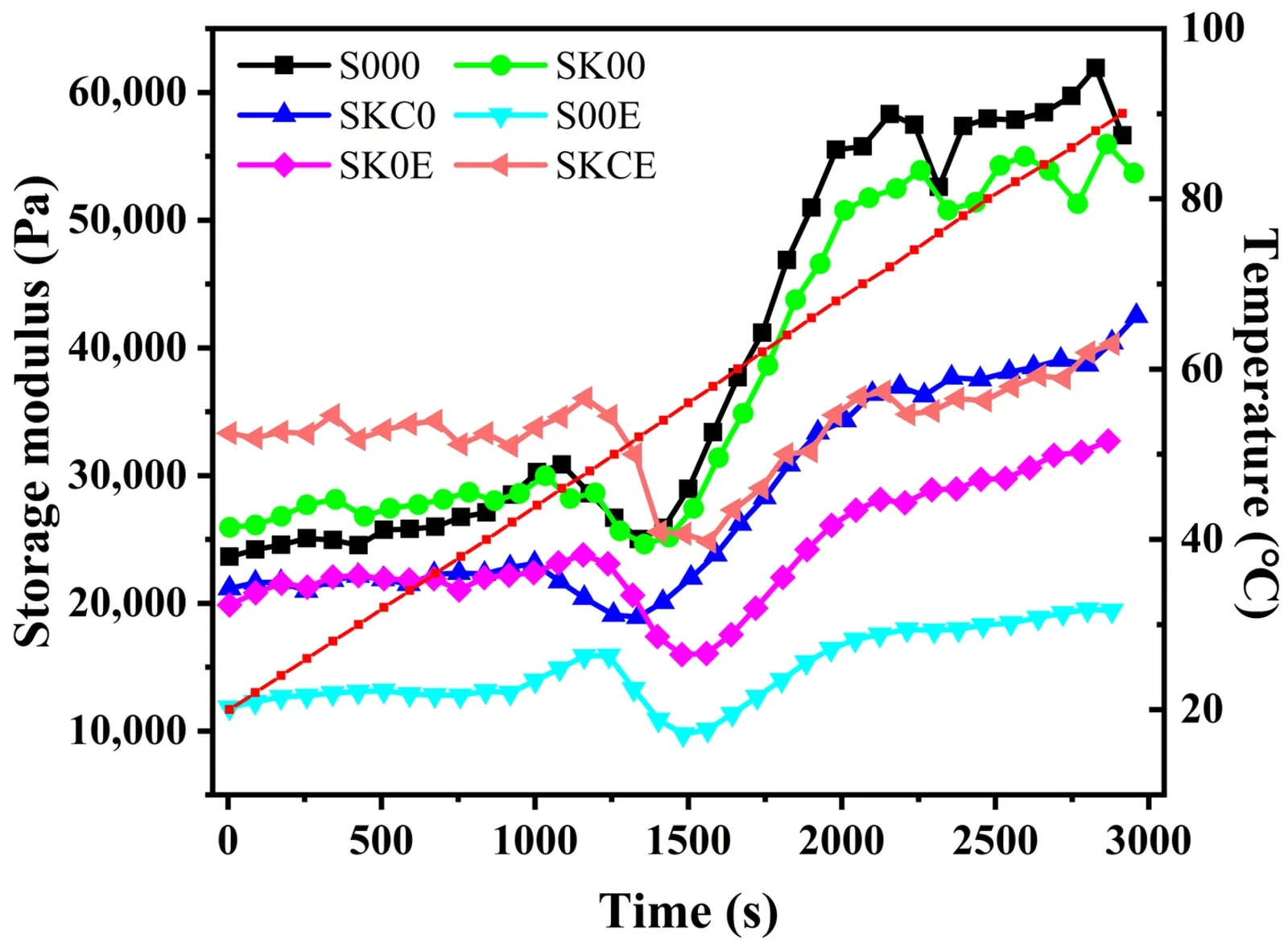
Changes in storage modulus (G′) of surimi gels during temperature sweep.

The storage modulus (G′), which reflects gel elasticity and protein gelling ability, increases with improved gel-forming capacity [41]. Effects of polysaccharides and emulsions on surimi were assessed via temperature sweep (20–90 °C). As shown in Figure 4, the G′ values of all samples exhibited three stages during heating. At 20–40 °C, G′ increased moderately, possibly owing to the formation of hydrogen bonds within the gel matrix. Rahman et al. reported that myosin head domains unfold within this temperature range, facilitating the formation of hydrogen bonds and interactions between myosin and polysaccharides [15]. In the 40–60 °C range, G′ decreased sharply, as hydrogen bonds were disrupted and the initial gel network loosened [42], compounded by the high proteolytic enzyme activity at this temperature [43]. During this stage, enzymatic degradation of MP may have outweighed the cross-linking effect of endogenous transglutaminase, thereby weakening the gel structure. From 60 to 90 °C, G′ gradually increased until equilibrium was reached. This increase is explained by thermal aggregation and cross-linking of the protein molecules, a process that facilitates the development of a robust and dense gel network [44]. Compared to the control (S000), samples with emulsions or polysaccharides had lower G′ values, probably due to weakened protein–protein cross-linking. Previous studies have shown that the addition of KGM to surimi can inhibit hydrogen-bond formation within myosin [15], resulting in a lower G′ value than that of the control (S000 vs. SK00). In S00E, the large oil droplets in the camellia oil emulsion increased the distance between protein molecules, interfered with intermolecular cross-linking and significantly reduced gelling ability [45]. This finding is consistent with the results of Li et al., who reported that emulsion incorporation enhanced the initial viscoelasticity of surimi but partially inhibited MP network formation during thermal gelation, ultimately decreasing G′ [46]. CRG can accelerate MP unfolding, promote myosin-head aggregation, and facilitate the formation of a compact MP network [10], which may explain the higher G′ observed after CRG addition in SK0E. In addition, KGM and CRG may have stabilized the emulsion by preventing oil-droplet aggregation and reducing droplet size [47]. These polysaccharides may also have contributed to the formation of a 3D network, thereby providing an elastic structure to the surimi gel.

### 2.5. Microstructure Analysis of Surimi Gels

**Figure 5:**
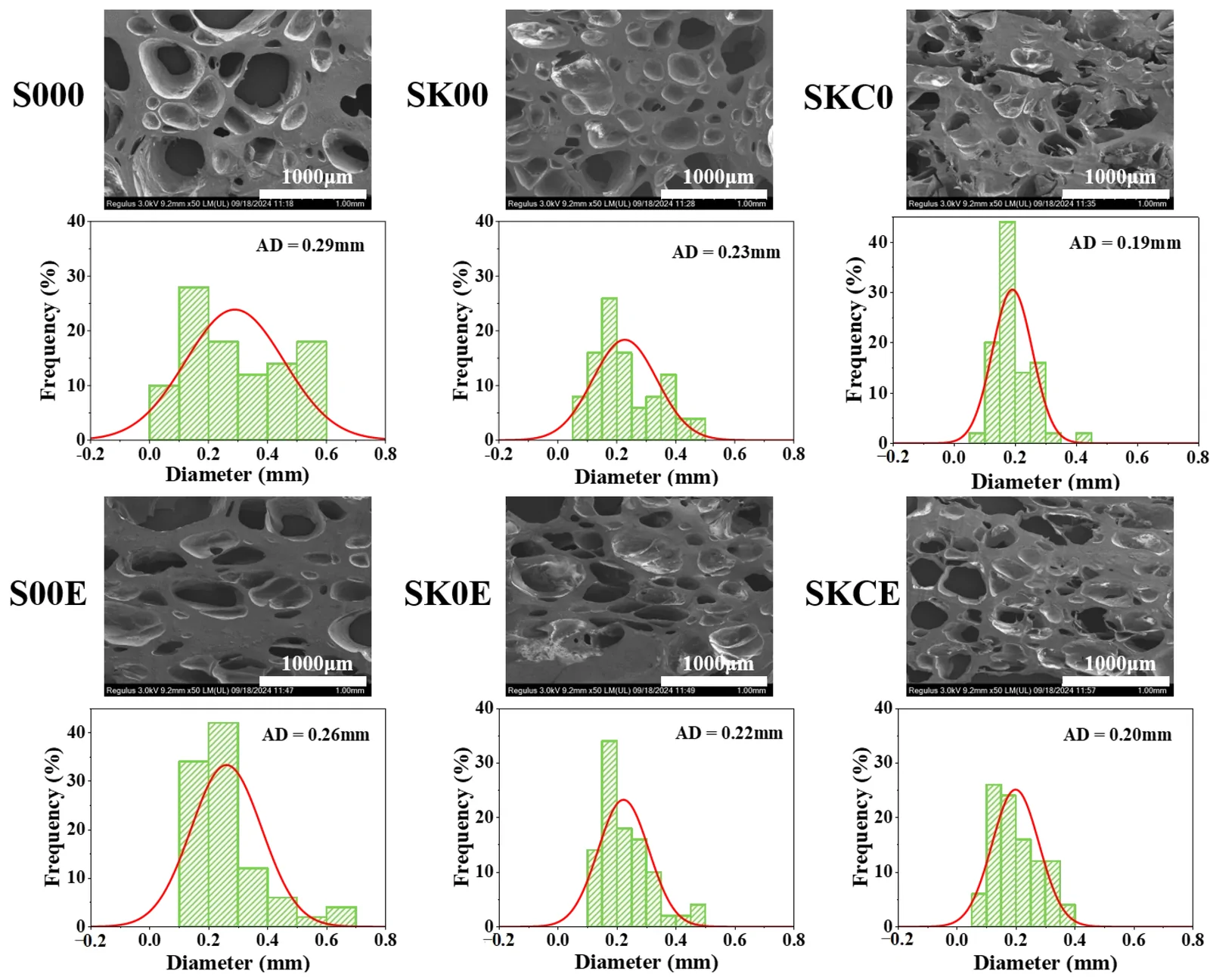
Microstructure, pore size and frequency distribution of surimi gels.

Figure 5 shows the SEM images of surimi gels. All samples exhibited a honeycomb-like microstructure, which is advantageous for water retention within the gels. The control sample (S000) presented large, uneven pores, a consequence of insufficient unfolding of MP [48], which negatively impacted WHC. Polysaccharide addition led to a reduction in pore size, more uniform distribution, and increased overall structural density. The pore-size analysis was consistent with the SEM observations (Figure 5). Owing to their hydrophilic and gel-forming properties, KGM and CRG filled the gaps within the surimi protein network, thereby promoting the formation of a more compact structure. During thermal gelation, the unfolding of protein and polysaccharide exposes hydrophilic groups and free sulfhydryl groups, which interact via disulfide bonds and noncovalent interactions, further densifying the matrix [49]. Incorporation of camellia oil emulsion alone did not markedly change pore number, but altered pore shape from round to elliptical (S00E). When polysaccharides were added to S00E, both the pore number and diameter decreased significantly, resulting in a denser network. SKCE displayed the densest and most ordered gel structure, indicating that the combination of polysaccharides and CAS-stabilized camellia oil emulsion synergistically enhanced the stability of the surimi gel. Previous studies have reported that KGM and CRG can form an interpenetrating network through non-covalent interactions, resulting in reduced droplet size and enhanced emulsion stability [47]. This process diminishes the barrier effect of the emulsion on MP and leads to improved gel network homogeneity. These findings are consistent with the observed results for WHC and cooking loss.

### 2.6. Sensory Analysis of Surimi Gels

**Figure 6:**
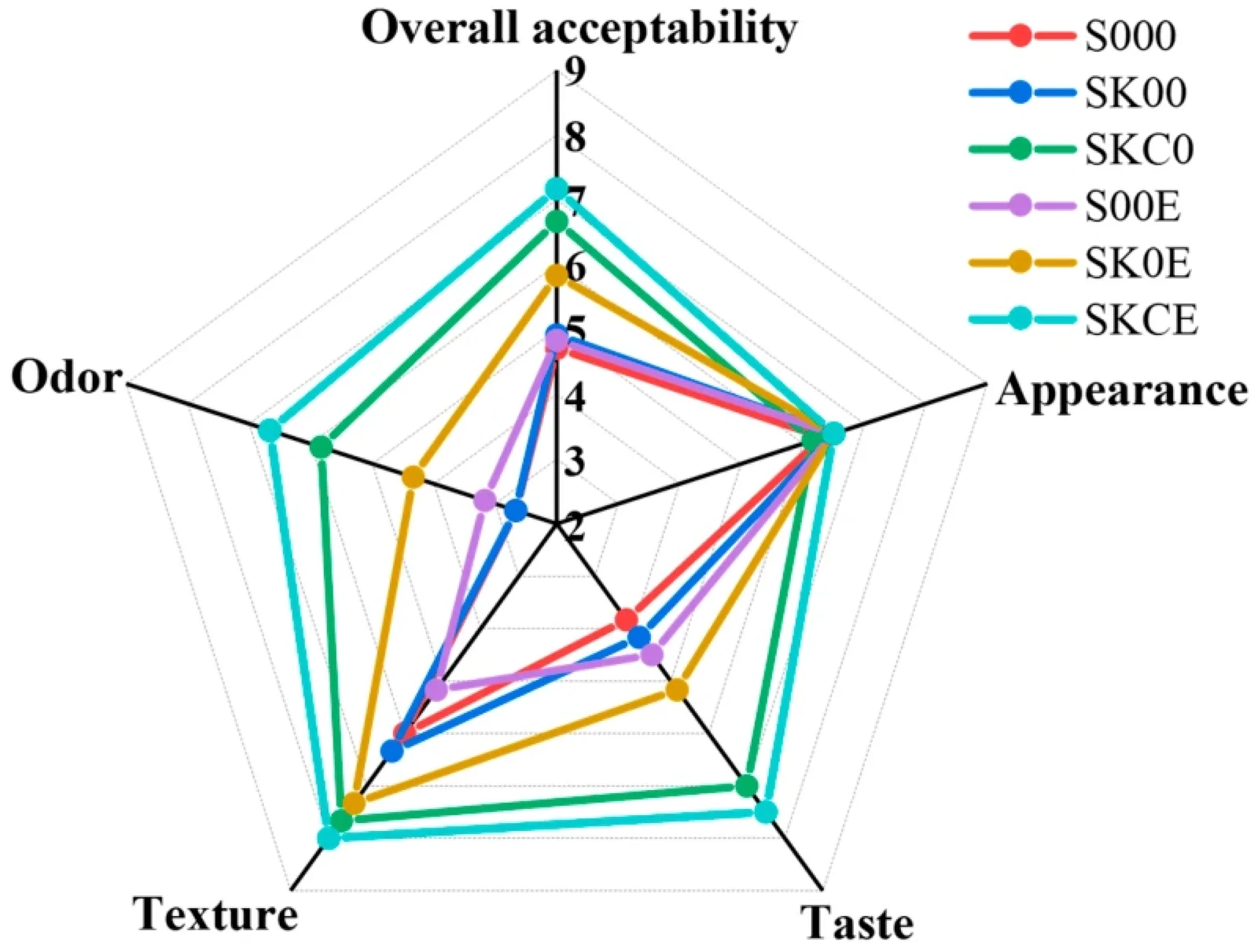
Sensory attributes of surimi gels.

The effects of CAS-stabilized camellia oil emulsion and polysaccharide-coupled emulsion on the appearance, odor, texture, taste, and overall acceptability of surimi gels are shown in Figure 6. All samples exhibited similar visual appearances. The control (S000) had relatively poor odor, as a result of lipid oxidation and MP denaturation during cooking. The introduction of camellia oil emulsion modestly improved odor and taste, while addition of KGM and CRG markedly enhanced odor, texture, taste, and overall acceptability. It has been reported that polysaccharides reduce protease activity, thereby limiting myofibrillar protein degradation and improving taste and texture of surimi [50]. Additionally, polysaccharide addition produces a denser gel network (Figure 5), thereby promoting the retention of volatile aroma compounds generated during the thermal processing of surimi. SKCE achieved the highest scores across all sensory attributes, indicating that the emulsion co-stabilized by KGM and CRG effectively enhances taste, preserves distinctive odor, and improves overall acceptability.

### 2.7. Freeze–Thaw Properties of Surimi Gels

**Figure 7:**
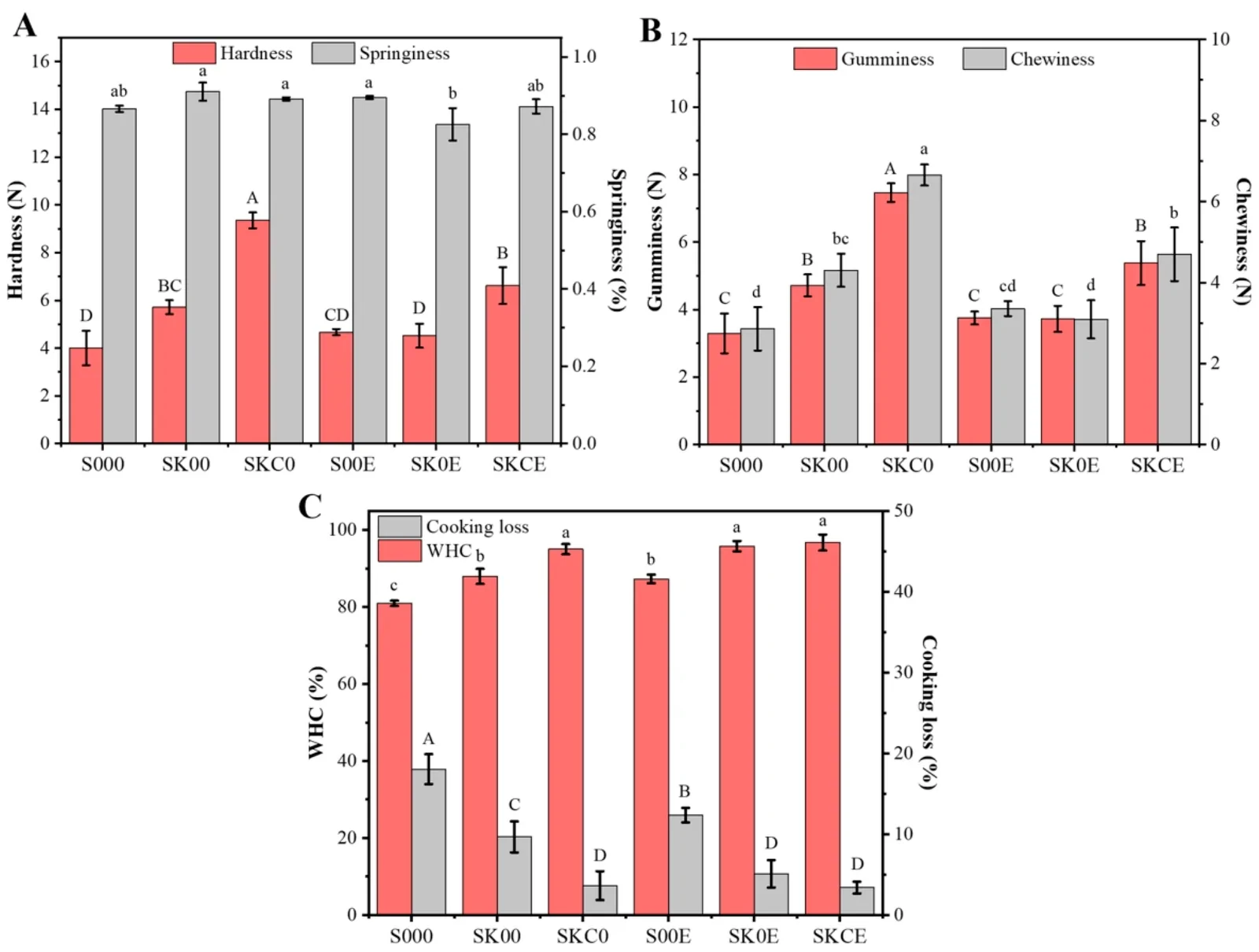
Textural properties (**A**,**B**), WHC, and cooking loss (**C**) of freeze–thaw treated surimi gels. Different capital and lowercase letters indicate significant differences (*p* < 0.05).

Surimi products are often subjected to freeze–thaw during transportation and storage, which can lead to quality deterioration and economic loss. Figure 7 presents the textural properties, cooking loss, and WHC of surimi gels following freeze–thaw treatment. The addition of polysaccharides, either alone or in combination with camellia oil emulsion, improved the textural properties of surimi gel after freeze–thaw treatment. These findings are consistent with the results observed prior to freeze–thaw treatment (Figure 2). Previous studies have demonstrated that polysaccharides promote the formation of uniform pore structures and facilitate ordered protein aggregation in surimi, resulting in denser, more homogeneous gel networks with finer pore structures. Furthermore, the hydrophilic nature of polysaccharides increases the viscosity of the unfrozen phase and reduces the free volume for solutes, thereby slowing ice crystal growth. KGM can also interact with ice crystals through hydrogen bonds, disrupting their structure. Therefore, the incorporation of polysaccharides into surimi can inhibit freeze–thaw-induced structural damage and enhance sample stability. Emulsions may further enhance freeze–thaw stability by acting as active fillers and influencing the crystallization behavior of both water and oil. Compared with the control (S000), the addition of emulsion alone (S00E) did not significantly affect the textural parameters of the surimi gels, whereas the addition of polysaccharides significantly increased all four parameters. Moreover, after freeze–thaw treatment, the samples containing both polysaccharides and emulsion exhibited a decreasing trend in their textural parameters compared with the samples containing polysaccharides alone (SKCE vs. SKC0; SK0E vs. SK00). These results indicate that polysaccharides have a greater influence on the freeze–thaw stability of surimi gels than the emulsion. Notably, the hardness of S00E decreased after freeze–thaw treatment, presumably due to emulsion leakage.

WHC and cooking loss serve as critical indicators for assessing the freeze–thaw stability of surimi gels. As shown in Figure 7C, trends in WHC and cooking loss were similar to those of textural properties. Samples with added polysaccharides, or both polysaccharides and emulsion, demonstrated improved WHC and reduced cooking loss after freeze–thaw. Compared to samples not subjected to freeze–thaw treatment (Figure 1), all samples exhibited decreased WHC and increased cooking loss, indicating that freeze–thaw treatment adversely affected the quality of surimi. However, the changes in WHC and cooking loss for SKC0 and SKCE were minor post-thaw. This indicates that KGM and CRG, alone or together with camellia oil emulsion, effectively enhance freeze–thaw stability surimi gels. In contrast, S00E exhibited the most significant changes in WHC and cooking loss before and after freeze–thaw treatment. Previous studies have shown that polysaccharides mitigate the adverse effects of freeze–thaw treatment on surimi gel quality by intervening water mobility and modifying the freezing rate. Furthermore, protein–polysaccharide co-stabilized emulsions enhance the dispersion of small droplets within the surimi gel, promoting the development of a dense and orderly 3D protein network [51]. These, in turn, contribute to improved surimi stability.

## 3. Conclusions

In this study, KGM and CRG were used in combination with CAS-stabilized camellia oil emulsion to enhance the comprehensive quality of freshwater surimi gels. In comparison to the individual addition of polysaccharides or emulsion, the combined application significantly elevates WHC, textural properties, freeze–thaw stability, and overall acceptability under controlled experimental settings, while reducing cooking loss. Microstructural analysis revealed that these multi-component interactions promoted the development of a denser, more ordered gel network, underlying these quality enhancements. This study provides preliminary laboratory-scale insights into the structural regulation of surimi gel systems by natural additives and offers a basis for developing high-quality surimi products. Before the formulation can be implemented in commercial-scale production, however, its practical feasibility must be systematically assessed, including ingredient costs and compatibility with existing processing methods.

## 4. Materials and Methods

### 4.1. Materials

KGM (Mw 1.4 × 10^6^ Da) was obtained from Hubei Yizhi Konjac Industry Co., Ltd. (Yichang, China). CRG was provided by Aladdin Biochemical Technology Co., Ltd. (Shanghai, China). CAS (protein ≥ 90%) and sodium chloride were sourced from Sinopharm Chemical Reagent Co., Ltd. (Shanghai, China). Frozen silver carp surimi was supplied by Honghu Jingli Aquatic Food Co., Ltd. (Jingzhou, China). Camellia oil was purchased from Henan Lvda Camellia Oil Co., Ltd. (Xinyang, China).

### 4.2. Preparation of Surimi Gels

First, a CAS-stabilized camellia oil emulsion was prepared as follows: CAS powder was dissolved in distilled water at a concentration of 1 wt% and stirred at 200 rpm for 3 h on a magnetic stirrer (CJJ78-1, Jinbiao District Xiyu New District Xinrui Instrument Factory, China), to obtain a homogeneous CAS solution. The CAS solution (aqueous phase) and camellia oil (oil phase, 10% (*v*/*v*)) were homogenized at 10,000 rpm for 3 min using an IKA Ultra-Turrax T25 homogenizer (IKA Works Co., Ltd., Guangzhou, China), resulting in the formation of a CAS-stabilized camellia oil emulsion. Frozen surimi was thawed at 4 °C, then processed in a chopper (QSJ-D03X1, Bear Electric Appliance Co., Ltd., Foshan, China) for 30 s to obtain coarse surimi. Subsequently, specified amounts of KGM and CRG were added as needed, followed by 2% sodium chloride. The moisture content was adjusted to 80% using either distilled water or the prepared CAS-stabilized camellia oil emulsion. The resulting mixture was chopped further for 2 min to produce homogenized surimi. The surimi mix was then transferred into plastic centrifuge tubes (50 mL, 35 mm diameter) and centrifuged at 4 °C and 1000× *g* for 5 min using a centrifuge (H1650-W, Hunan Xiangyi Laboratory Instrument Development Co., Ltd., Xiangtan, China) to remove trapped air bubbles. The surimi samples were then subjected to a two-stage thermal setting: incubation at 40 °C for 30 min, followed by heating at 90 °C for another 30 min in a water bath (HH-2, Changzhou Saipu Experimental Instrument Factory, Changzhou, China). After cooling to ambient temperature, the resulting gels were stored at 4 °C for subsequent analyses. The detailed composition of each surimi gel sample is provided in Table 1. The concentrations of KGM, CRG, and the CAS-stabilized camellia oil emulsion are based on previous reports [52].

### 4.3. Freeze–Thaw Treatment of Surimi Gels

The surimi mixture was centrifuged at 1000× *g* for 5 min to eliminate air bubbles. The samples were then subjected to a freeze–thaw cycle according to the method described previously [53], with slight modifications. Briefly, the samples were frozen at −20 °C for 22 h and subsequently thawed at 25 °C for 2 h. The thawed samples were subsequently heated following the procedure in Section 4.2 to yield freeze–thaw treated surimi gels.

### 4.4. Cooking Loss Determination

Centrifuge tubes were pre-weighed, and approximately 2 g of surimi mixture was added to each tube and weighed again. Samples were then heated following the procedure in Section 4.2. After cooling to room temperature, the samples were equilibrated at 4 °C for 2 h. Excess surface moisture was removed with filter paper, and the samples were weighed. Cooking loss was calculated as follows [4]:

(1) Cooking loss (%) = (m_1_ − m_2_)/(m_1_ − m_0_) × 100

where m_0_ is the weight of the empty centrifuge tube, m_1_ is the weight before cooking, and m_2_ is the weight after cooking.

### 4.5. Water Holding Capacity (WHC) Measurement

The WHC was determined using a previous method [10]. Approximately 2 g of surimi gel was placed in a centrifuge tube and centrifuged at 8000× *g* for 10 min. Moisture on the gel surface was removed with filter paper, and the sample was weighed. The WHC was calculated as follows:

(2) WHC (%) = (M_2_ − M_0_)/(M_1 −_ M_0_) × 100

where M_0_ is the weight of the empty centrifuge tube, M_1_ is the weight before centrifugation, and M_2_ is the weight after centrifugation.

### 4.6. Texture Profile Measurement

Surimi gels, equilibrated to room temperature, were cut into cylinders with a uniform height (30 mm diameter). Textural properties were measured using a texture analyzer (TMS-Pro 3000, FTC, Sterling, VA, USA) equipped with a 25 mm cylindrical probe. The testing parameters were set as follows: test speed 20 mm/min, compression ratio 20%, interval time 5 s, and trigger force 0.1 N.

### 4.7. Fourier Transform Infrared (FTIR) Spectroscopy Measurement

FTIR analysis was conducted using a Perkin–Elmer spectrometer (Waltham, MA, USA). Gel samples were sectioned into thin slices and subsequently subjected to freeze-drying for 72 h. Approximately 5 mg of freeze-dried sample was mixed with potassium bromide and pressed into a pellet before scanning in the wavenumber range of 4000–400 cm^−1^ [17]. Protein secondary structures were analyzed using Ominic 8.2 software.

### 4.8. Rheological Measurement

A DHR-2 rheometer (TA Instruments, New Castle, DE, USA) was used to assess the storage modulus of surimi gels during temperature sweep. Samples were coated with silicone oil and loaded between parallel plates with diameter of 20 mm (1 mm gap). After 2 min equilibration, the gels were subjected to a temperature sweep from 20 °C to 90 °C (2 °C/min) [44]. The scanning frequency was 1 Hz, with a constant stress of 1 Pa.

### 4.9. Scanning Electron Microscope (SEM) of Surimi Gels

Surimi gel slices were freeze-dried, fixed using conductive adhesive, and sputter-coated with platinum [17]. Microstructural imaging was performed using a scanning electron microscope (Regulus 8220, Hitachi, Japan) at magnifications of 50×, using an acceleration voltage of 3 kV.

Pore size and its frequency distribution were determined from SEM images using Nano Measurer software (Version 1.2.5, Hitachi, Japan). Three replicate analyses were performed for each sample. In each analysis, three SEM images of the same sample were analyzed, 50 pores were measured manually, and the mean pore size was calculated.

### 4.10. Sensory Evaluation

Sensory evaluation was conducted in accordance with GB/T 39625–2020 (National Standardization Administration Committee, 2020, Beijing, China) and the method described by Sun et al. [54]. Twenty postgraduate students with relevant academic backgrounds and at least five months of sensory evaluation experience were recruited to serve as panelists. Panelists were selected based on their interest in sensory evaluation, taste sensitivity, and preference for surimi products. The samples were evaluated for appearance, taste, texture, odor, and overall acceptability in a dedicated sensory evaluation room equipped with individual booths. The room was maintained at 25 ± 1 °C and 50–55% relative humidity. Fluorescent lighting was directed vertically onto the sample surfaces to minimize shadows. Detailed evaluation criteria are provided in Supplementary Table S1.

### 4.11. Statistical Analysis

All experiments were performed in quintuplicate. Data were expressed as mean ± standard deviation (SD). Before conducting a one-way analysis of variance (ANOVA), all datasets were assessed for normality and homogeneity of variance. Statistical analyses were conducted by one-way ANOVA followed by Duncan’s test at a significance level of *p* < 0.05 using SPSS 25.0 (IBM, Chicago, IL, USA).

## Figures and Tables

**Table 1:** The content of KGM, CRG, and emulsion added in surimi gels.

Samples	KGM (*w*/*w*_0_)	CRG (*w*/*w*_0_)	CAS-Stabilized Camellia Emulsion (*w*/*w*_0_)
S000	0	0	0
SK00	0.6%	0	0
SKC0	0.6%	1%	0
S00E	0	0	8%
SK0E	0.6%	0	8%
SKCE	0.6%	1%	8%

Note: *w* represents the mass of KGM, CRG or CAS-stabilized camellia emulsion. *w*_0_ represents the mass of surimi. All samples contained 2% sodium chloride and had a moisture content of 80%.

## Data Availability

The data presented in this article/the Supplementary Materials are available from the corresponding author on request.

## Supplementary Materials

The following supporting information can be downloaded at https://www.mdpi.com/article/10.3390/gels12100907/s1.

## References

[1] Walayat N., Blanch M., Moreno H.M. (2025). Surimi and Low-salt Surimi Gelation: Key Components to Enhance the Physicochemical Properties of Gels. Gels.

[2] Wang Z., Liu S., Yang W., Geng J.-T., Huang T., Wei H., Qiao Z., Jia R. (2024). Ovalbumin/Sodium Alginate Pickering Emulsion: Structural Characteristics and Its Contribution to Enhancing the Gel Properties of Hairtail (*Trichiurus haumela*) Surimi. Food Chem..

[3] Yan W., Ouyang Z., Luo X., Xiao R., Liao S., Jiang F., Li Y., Xiong S., Yin T., Zhu X. (2025). Effect of Carboxymethyl Konjac Glucomannan on the Gel Properties of Silver Carp Surimi: A study on the Regulatory Mechanism of Substitution Degree. Foods.

[4] Sun Y., Zhao M., Shi H., Liu Z., Zhang X., Xia G., Yu G. (2025). Improvement of Freeze-thaw Stability of Lipid-enhanced Skipjack Tuna (*Katsuwonus pelamis*) Surimi Gels via Pickering High Internal Phase Emulsions. Food Res. Int..

[5] Yang R., Juma N.S., Zhao Y., Zheng B., Xu Y., Gao Y., Jia R., Gao P., He Y. (2025). Factors Influencing Surimi Gelling Properties and Natural Additive–based Gel Fortification Strategies: A Review. Compr. Rev. Food Sci. Food Saf..

[6] Nie J., Xue C., Xiong S., Yin T., Huang Q. (2024). Comparative Analysis of Soluble and Insoluble Dietary Fiber on Improving the Gelation Performance and Fishy Odors of Silver Carp Surimi. Int. J. Biol. Macromol..

[7] Sun X., Li Q., Ding N., Liang S., Mubango E., Zheng Y., Yu Q., Dai R., Tan Y., Luo Y. (2024). Cryoprotective Effect of Fistular Onion Stalk Polysaccharide on Frozen Surimi Derived from Bighead Carp: Physicochemical Properties and Gel Quality During Storage. Food Hydrocoll..

[8] Chen M., Yang L., Wang J., Jiang J., Feng M., Wu W., Chen S., Wang L., Shi L. (2025). Towards Healthier Surimi: Quality and Nutritional Benefits of Flaxseed Gum as a Fat Replacer in Grass Carp Surimi Gels. Food Chem..

[9] Mi H., Li Y., Wang C., Yi S., Li X., Li J. (2021). The Interaction of Starch-gums and Their Effect on Gel Properties and Protein Conformation of Silver Carp Surimi. Food Hydrocoll..

[10] Cao C., Zhang J., Kong B., Sun F., Zhang H., Liu Q. (2024). Underlying the Mechanisms of Incorporation of κ-carrageenan on the Formation of Low-salt Myofibrillar Protein Gels During Heating Process: Perspective on the Dynamic Changes of Protein Structures and Molecular Interactions. Food Hydrocoll..

[11] Lan H., Chen L., Wang Y., Lu M., Chen B., Ai C., Teng H. (2023). Effect of κ-carrageenan on Saltiness Perception and Texture Characteristic Related to Salt Release in Low-salt Surimi. Int. J. Biol. Macromol..

[12] Wei Q.-J., Zhang W.-W., Wang J.-J., Thakur K., Hu F., Khan M.R., Zhang J.-G., Wei Z.-J. (2024). Effect of κ-Carrageenan on the Quality of Crayfish Surimi Gels. Food Chem. X.

[13] Huang C., Blecker C., Wei X., Xie X., Li S., Chen L., Zhang D. (2024). Effects of Different Plant Polysaccharides as Fat Substitutes on the Gel Properties, Microstructure and Digestion Characteristics of Myofibrillar Protein. Food Hydrocoll..

[14] Liu S., Hu L., Xiang Y., Liu Q., Zhao X., Shao J.-H. (2025). Interactions Between Myofibrillar Protein and Konjac Glucomannan at Critical Thermal Phase Transition Temperatures: Aggregation, Cross-linking, and Protein Conformational Changes. Food Res. Int..

[15] Rahman Z., Zhang S., Khan A., You J., Liu R., Huang Q., Ma H., Benjakul S., Yin T. (2025). Effects of Modified KGM on the Gelling Capability of Fish Protein: Novel Insights into Hooking Effect. Food Chem..

[16] Shi H., Zhang M., Liu X.-C., Yao X., Wang W., Zheng J., Tomasevic I., Sun W. (2024). Improved Qualities of Salt-reduced Tilapia Surimi by Adding Konjac Glucomannan: Insight into the Edible Traits, Gel Properties and Anti-freezing Ability. Food Hydrocoll..

[17] Zhang L., Ning Y., Wei Y., Xin Y., Shah B.R., Du H., Zou J., Xu W. (2025). Effect of K2CO3 on Konjac Glucomannan/κ-Carrageenan-Based Camellia Oil Pickering Emulsion Gel for The Development of Fat Analogs. Int. J. Biol. Macromol..

[18] Wu G., Yin Y., Li C., Hao R., Zang J., Kang M., Luo D., Xu W. (2024). Sustained Release Behavior of Lysozyme Based on Konjac Glucomannan/κ-Carrageenan Composite Hydrogels. LWT.

[19] Ren Z., Huang X., Zhao Y., Shi L., Yang S., Jin R., Lin R., Liu S., Liu Z. (2025). Effect of Myosin-kappa Carrageenan Pickering Emulsions on the Quality of Bighead Carp Surimi Gels. Food Hydrocoll..

[20] Wang Z., Liu S., Geng J.-T., Yang W., Huang T., Wei H., Qiao Z., Jia R. (2025). Insights into the Mechanism of Ovalbumin-sodium Alginate Pickering Emulsions on the Gel and Emulsification Properties of Hairtail (*Trichiurus haumela*) Myofibrillar Protein. J. Agric. Food Res..

[21] Zhao Q., Zheng B., Li J., Cheong K.L., Li R., Chen J., Liu X., Jia X., Song B., Wang Z. (2024). Emulsion-filled Surimi Gel: A Promising Approach for Enhancing Gel Properties, Water Holding Capacity, and Flavor. Trends Food Sci. Technol..

[22] Liu C., Liang B., Li Z., Liu Y., Yi S., Li J., Ye B., Li X., Lu Y., Liu S.-Q. (2025). Formation and Network Properties of Emulsified Surimi Gels: Effects of Sodium Starch Octenylsuccinate Stabilized Silver Carp Oil Pickering Emulsions. LWT.

[23] Li J., Shen J., Shen W., Jin W., Guo C., Xiong S., Hu Y. (2026). Comparative Study on the in Vitro Digestive Characteristics of Surimi Gels Cross-linked by EGCG and/or TGase. Food Chem. X.

[24] Liu C., Ye B., Yi S., Li X., Xu Y., Xia W. (2025). Improvement of The Rheological Properties and Freeze-thawing Stability of Pickering Emulsions Filled Emulsified Surimi Gels: Effects of The Interfaces of Surimi Particles and Konjac Glucomannan. Food Hydrocoll..

[25] Zhou Y., Lu S., Chen H., Xu Y., Yin F., Zhang B., Pei Z., Zhang L., Zhou D., Li C. (2025). Potential Freezing Resistance of Collagen Peptide-fish Oil High Internal Phase Emulsion: Enhancing Freeze-thaw Stability of 3D-printed Surimi. Food Hydrocoll..

[26] Mi H., Liang S., Chen J., Li X., Li J. (2024). Effect of Starch-based Emulsion with Different Amylose Content on The Gel Properties of *Nemipterus virgatus* Surimi. Int. J. Biol. Macromol..

[27] Xu Y., Lv Y., Zhao H., He X., Li X., Yi S., Li J. (2022). Diacylglycerol Pre-emulsion Prepared Through Ultrasound Improves the Gel Properties of Golden Thread Surimi. Ultrason. Sonochem..

[28] Wang Y., Yu G., Liu Z., Shi H., Zhang X., Zhao Y., Wang J., Xia G. (2025). Effects of Sodium Caseinate/Tannic Acid Stabilization of High Internal Phase Fish Oil Emulsions on Surimi Gel Properties and 3D Printing Quality. LWT.

[29] Gao L., Jin L., Liu Q., Zhao K., Lin L., Zheng J., Li C., Chen B., Shen Y. (2024). Recent Advances in The Extraction, Composition Analysis and Bioactivity of Camellia (*Camellia oleifera* Abel.) Oil. Trends Food Sci. Technol..

[30] Xu W., Xiong Y., Li Z., Luo D., Wang Z., Sun Y., Shah B.R. (2020). Stability, Microstructural and Rheological Properties of Complex Prebiotic Emulsion Stabilized by Sodium Caseinate with Inulin and Konjac Glucomannan. Food Hydrocoll..

[31] Xu W., Zang J., Du S., Zhang C., Jia Y., Zhang L., Zhang X. (2025). Sodium Caseinate Stabilized Camellia Oil Emulsion with Konjac Glucomannan as Fat Replacer in Low-alcohol Ice Cream. LWT.

[32] Canti M., Martawidjaja K.L. (2024). Physicochemical and Sensory Properties of Kamaboko Produced from Asian Seabass Surimi-catfish Protein Isolate (*Clarias gariepinus*). Meas. Food.

[33] Xie Y., Yu X., Wang Z., Yu C., Prakash S. (2022). The Synergistic Effects of Myofibrillar Protein Enrichment and Homogenization on The Quality of Cod Protein gel. Food Hydrocoll..

[34] Li X., Fan M., Huang Q., Zhao S., Xiong S., Yin T., Zhang B. (2022). Effect of Micro-and Nano-starch on The Gel Properties, Microstructure and Water Mobility of Myofibrillar Protein from Grass Carp. Food Chem..

[35] Sun Y., Sun J., Zhao M., Zhao M., Shi H., Liu Z., Zhang X., Xia G. (2025). Enhancing Gel and 3D Printing Performance of Lipid-enhanced Skipjack Tuna (*Katsuwonus pelamis*) Surimi via Pickering High Internal Phase Emulsion. Food Res. Int..

[36] Lv Y., Sun X., Jia H., Hao R., Jan M., Xu X., Li S., Dong X., Pan J. (2023). Antarctic Krill (*Euphausia superba*) Oil High Internal Phase Emulsions Improved the Lipid Quality and Gel Properties of Surimi Gel. Food Chem..

[37] Geng X., Zhao Y., Zhao N., Zhu Q., Zhang M. (2023). Quality Characteristics and Gastrointestinal Fate of Low Fat Emulsified Sausage Formulated with Konjac Glucomannan/Oat β-Glucan Composite Hydrogel. Int. J. Biol. Macromol..

[38] Liu S., Wang Z., Yang W., Huang T., Geng J.-T., Wei H., Gao Y., Jia R. (2025). Carboxymethyl Chitosan-assisted Formation of Stable Soy Protein Isolate Emulsions and Their Impact on The Properties of Squid (*Dosidicus gigas*) Surimi Gel. Food Hydrocoll..

[39] Zhu J., Dai H., Yuan C., Tang S., Ma L., Tan X., Wang H., Zhang Y. (2026). Enhancing Surimi Structure by Lycopene-Fennel Extract Pickering Emulsion: Application in 3D Printing Analog Beef with Dynamic Color and Flavor Retention. Food Res. Int..

[40] Zhuang X., Jiang X., Zhou H., Chen Y., Zhao Y., Yang H., Zhou G. (2020). Insight Into the Mechanism of Physicochemical Influence by Three Polysaccharides on Myofibrillar Protein Gelation. Carbohydr. Polym..

[41] Chen M., Sun Z., Ma A., Shi G., Xiong G., Qiao Y., Chen S., Wu W., Liu J., Tu Z. (2025). Enhancing Surimi Gel Properties in Silver Carp with Innovative Ultra-micro Crayfish Shell Powder. Food Chem..

[42] Piao X., Huang J., Sun Y., Zhao Y., Zheng B., Zhou Y., Yu H., Zhou R., Cullen P.J. (2023). Inulin for Surimi Gel Fortification: Performance and Molecular Weight-dependent Effects. Carbohydr. Polym..

[43] Lan H., Chen L., Zhou R., Bai S., Ai C., Teng H. (2025). Gel Properties, Phase Behavior and Flavor of Double-network Surimi Gel: Interactions, Performance and Mechanisms. Food Chem..

[44] Zhang P., Zheng M., Hou G., Zhou F., Deng K., Miao S., Zhang L., Zheng B. (2024). Enhanced Gelation Properties and Saltiness Perception of Low-salt Surimi Gel with Psyllium Husk Powder. J. Agric. Food Chem..

[45] Wang H., Zhang J., Xu Y., Mi H., Yi S., Gao R., Li X., Li J. (2023). Effects of Chickpea Protein-stabilized Pickering Emulsion on The Structure and Gelling Properties of Hairtail Fish Myosin Gel. Food Chem..

[46] Li J., Jiang X., Yang L., Wang K., Ma H., Wang D., Shi W. (2026). Development and Characterization of Glycosylated Chickpea Protein Isolate Oleogel Emulsion: A Fat Substitute for Application in Oil-Fortified Surimi. Food Res. Int..

[47] Zhang X., Ning Y., Chai L., Yin Y., Luo D., Xu W. (2024). Physicochemical Properties and in Vitro Digestive Behavior of Astaxanthin Loaded Pickering Emulsion Gel Regulated by Konjac Glucomannan and κ-Carrageenan. Int. J. Biol. Macromol..

[48] Wu D., Xiong J., Li P., Zhang Y., Li F., Yin T., Huang Q. (2024). Dual Enhancement Effects of Different Yeast Extract on Gel Properties and Saltiness Perception of Low-salt Surimi Gel from Silver Carp. Food Hydrocoll..

[49] Wu S. (2016). Effect of Pullulan on Gel Properties of *Scomberomorus niphonius* Surimi. Int. J. Biol. Macromol..

[50] Mao Y., Zhang Y., Li T., Chen Y., Wang Z., Jin W., Shen W., Li J. (2025). Insight into The Mechanism of Gel Properties, Microstructure and Flavor of Surimi Gels Improved by Wheat Bran with Different Particle Sizes. Food Res. Int..

[51] Xu Y., Yu J., Xue Y., Xue C. (2023). Enhancing Gel Performance of Surimi Gels via Emulsion Co-stabilized with Soy Protein Isolate and κ-Carrageenan. Food Hydrocoll..

[52] Chen W., Wu G., Zhang L., Zhang L., Shah B.R., Xu W. (2025). Comparative Study on the Freeze-Thaw Stability of Sodium Caseinate Emulsion-Filled Konjac Glucomannan/κ-Carrageenan Composite Gels. Gels.

[53] Xu Y., Lu Q., Wang H., Febrisiantosa A., Hu Y., Zhang L., Chen X., Zhu L., Li C. (2026). Enhancing the Freeze-Thaw Stability of 3D-printed Surimi Gels using High Internal Phase Pickering Emulsions with “Fried Egg-Like” Interfaces. Food Hydrocoll..

[54] Guo R., Xiong J., Li P., Ma C., Zhao X., Cai W., Kong Y., Huang Q. (2024). Emulsified sausages with yeast protein as an Animal Fat Replacer: Effects on Nutritional Composition, Spatial Structure, Gel Performance, and Sensory Quality. Meat Sci..

