# Supplementary material for: Surimi Gel Quality Enhanced by κ-Carrageenan/Konjac Glucomannan and Sodium Caseinate-Stabilized Emulsion
Source: Gels. 2026 Oct 8;12(10):907. doi: 10.3390/gels12100907 (PMC13704134; doi:10.3390/gels12100907)
Supplement: Supplementary file 1 [file gels-12-00907-s001.zip › gels-4567763-supplementary.pdf]

Supplementary Table S1 Sensory evaluation rules for surimi gel

Project	Score	Evaluation standard
Appearance	9-10	Glossy, pure white and bright, appetizing
	5-8	Slightly glossy, relatively pure white and bright
	2-4	Slightly inferior color and luster
	0-1	Dull color and luster
Taste	9-10	Desirable elasticity, moderate chewiness, well-balanced taste
	5-8	Adequate elasticity, slightly hard or soft texture
	2-4	Limited elasticity, hard or soft texture
	0-1	Poor elasticity, difficult to chew or lacks necessary integrity
Texture	9-10	Uniform and dense section, almost no air pockets
	5-8	Relatively dense section, small and uniform air pockets
	2-4	Inferior section, air pockets of varying sizes
	0-1	Loose and coarse section, large and uneven air pockets
Odor	9-10	Strong fish fragrance, no fishy odor
	5-8	Moderate fish fragrance, light fishy odor
	2-4	Weak fish fragrance, moderate fishy odor
	0-1	No fish fragrance, strong fishy odor or off-flavor
Overall Acceptance	9-10	Excellent
	5-8	Acceptable
	2-4	Difficult to accept
	0-1	Completely unacceptable
